# Comparison of self-reported and job-exposure matrix assessed workplace inhalant exposures and their association with obstructive airways disease

**DOI:** 10.1038/s41598-025-95923-w

**Published:** 2025-04-23

**Authors:** Lukas S Damerau, Robert Herold, Alexandra M Preisser, Alena Haack, Elina L Petersen, Benjamin Waschki, Christina Magnussen, Peter Koch, Albert Nienhaus, Volker Harth, Hanno Hoven

**Affiliations:** 1 Institute for Occupational and Maritime Medicine (Zfam), University Medical Center Hamburg-Eppendorf (UKE), 20459 Hamburg, Germany; 2 Epidemiological Study Center, University Hospital Hamburg-Eppendorf (UKE), Hamburg, Germany; 3 Department of Cardiology, Heart & Vascular Center Hamburg, University Medical Center Hamburg-Eppendorf (UKE), Hamburg, Germany; 4 Airway Research Center North (ARCN), Pulmonary Research Institute at LungenClinic Grosshansdorf, Grosshansdorf, Germany; 5 Hospital Itzehoe, Pneumology, Itzehoe, Germany; 6 Center for Population Health Innovation, University Heart and Vascular Center Hamburg, University Medical Center Hamburg-Eppendorf (UKE), Hamburg, Germany; 7 German Center for Cardiovascular Research, Partner Site Hamburg/Kiel/Lübeck, Hamburg, Germany; 8 Institute for Health Services Research in Dermatology and Nursing (IVDP), Centre for Epidemiology and Health Services Research for Healthcare Professionals (Cvcare), University Hospital Hamburg-Eppendorf (UKE), Hamburg, Germany; 9 Department for Occupational Medicine, Hazardous Substances and Health Sciences (AGG), German Social Accident Insurance for the Health and Welfare Services (BGW), Hamburg, Germany; 10 Centre for Global Health Inequalities Research (CHAIN), Department of Sociology and Political Science, Norwegian University of Science and Technology (NTNU), Trondheim, Norway

**Keywords:** COPD, GOLD, GLI, JEM, VGDF, Occupational health, Chronic obstructive pulmonary disease, Epidemiology

## Abstract

Chronic obstructive pulmonary diseases (COPD) are major public health concerns, with occupational exposure to vapors, gases, dusts, and fumes (VGDF) often overlooked as risk factors. This study investigates discrepancies between self-reported and Job-Exposure Matrix (JEM)-assessed exposures in chronic airway obstruction (AO), highlighting the importance of robust assessment methods. Data from the Hamburg City Health Study (2016–2020) were analyzed, contrasting self-reported VGDF exposure with Airborne Chemical Exposure (ACE) JEM assessments. COPD definition followed GOLD (Global Initiative for Chronic Obstructive Lung Disease) and GLI (The Global Lung Function Initiative) criteria for an AO. Inter-rater reliability was calculated using Cohen’s Kappa. Logistic regression models analyzed VGDF-AO associations, adjusting for confounders. Among 4,811 participants assessed with GOLD criteria, 3,545 met GLI criteria; inter-rater reliability between self-reported and JEM-based exposure was modest (Kappa = 0.29). Awareness of exposure varied between skill levels. Self-reported VGDF exposure was associated with GLI-defined AO (OR 1.48, 95% CI: 1.11–1.96), but not with GOLD-defined AO. JEM assessments did not show an association for either criterion. Discrepancies exist between self-reported and JEM-assessed VGDF exposures, as well as between GOLD and GLI criteria. Enhanced assessment strategies are needed to accurately assess occupational health risks related to COPD.

## Introduction

Chronic obstructive pulmonary disease (COPD) and other chronic diseases with airway obstruction (AO) are a major global health concern and has recently become the third most common cause of mortality globally^1^. While smoking is still the dominant risk factor for developing chronic diseases with AO^2–4^, occupational exposure to inhalable noxious agents accounts for at least 15% of all COPD cases^5–8^. Among individuals who have never smoked, such occupational exposures are responsible for as many as 31% of COPD cases^9^. Despite the significant contribution of occupational hazards to COPD development, these exposures are often undiagnosed in clinical practice^10^. Accurate assessment methods are crucial for identifying and mitigating these risks. The complexity of workplace exposure to vapors, gases, dusts, and fumes (VGDF) is a notable factor in the development of COPD. These inhalable agents are prevalent in several occupational environments, such as agriculture, manufacturing, and mining. In these settings, exposure to organic dusts, chemical vapors, and other harmful substances is often unavoidable, posing significant respiratory health risks. For instance, agricultural workers are frequently exposed to organic dusts, while manufacturing and mining workers encounter a range of chemical vapors and inorganic dusts, leading to an elevated incidence of COPD^11,12^. The chronic and often prolonged nature of this exposure significantly contributes to respiratory complications and COPD development. The risk is further intensified in the presence of preexisting respiratory conditions or smoking habits, as these factors can synergistically worsen lung function decline and inflammation, which are hallmark features of COPD^13^.

Occupational exposure to VGDF is measured in various ways with no gold standard. While an individual workplace risk assessment by an expert may be preferable, it is often only feasible for small studies. Thus, the two most commonly used methods in larger population-based cohorts are (1) self-reporting based on interviews or questionnaires and (2) Job-Exposure-Matrices (JEM).

Self-reported exposures provide insights into an individual’s perception and awareness of workplace conditions and risks. However, this method is vulnerable to certain biases that may compromise the accuracy of exposure assessments. Specifically, self-reporting is susceptible to recall bias, where individuals may not accurately remember past exposures. Additionally, social desirability bias, where respondents might alter their answers to align with perceived social expectations, can further skew the data^14,15^. The effectiveness of self-reporting hinges significantly on employees’ awareness of the hazards associated with VGDF. A high level of awareness can lead to more precise reporting and proactive mitigation measures. Conversely, limited awareness or underestimation of risks could result in underreporting and an inaccurate classification of exposure levels, thereby undermining the reliability of the collected data^16^.

In contrast, JEMs are based on standardized exposure evaluations by occupational health experts, where each job title of occupational classification schemes (e.g., The Standard Occupational Classification (SOC 2000^17^)) is assigned a specific exposure level. This method reduces the risk of recall bias associated with self-reporting and provides a more objective assessment of workplace exposures. Using JEMs is usually more effortful than using self-reported data because every individual must be given an occupational classification code, which is often assigned manually to the individuals’ job title but can be enhanced to be done automatically do a certain degree^18,19^. Yet, JEMs often oversimplify complex exposure scenarios, failing to capture nuanced details of an individual’s work history, specific workplaces, and tasks^14,15,20^.

This study aims to investigate the link between occupational exposure to VGDF and AO, specifically focusing on evaluating and comparing two distinct exposure assessment methods: self-reporting and JEM. Additionally, it will assess the outcomes using two different criteria, the Global Initiative for Chronic Obstructive Lung Disease (GOLD)^21^and the Global Lung Function Initiative (GLI)^22^ standards. By contrasting these methodologies, this study seeks to identify any notable discrepancies in accuracy and reliability in detecting occupational contributors to diseases with chronic bronchial obstruction like COPD. The findings are anticipated to contribute significantly to refining occupational health and safety interventions and policies, ensuring that they are grounded in the most reliable and precise exposure assessment techniques available.

## Methods

### Study design

Cross-sectional data from the “Hamburg City Health Study” (HCHS) were used^23^. Residents of Hamburg aged between 45 and 74 years were deemed eligible and selected at random by the registration office. They were invited to a single study center, where they underwent various health assessments conducted by trained study nurses. In addition, participants were asked to fill out detailed questionnaires, providing further insights into their health status and lifestyle factors. Data were collected between February 2016 and December 2020. The local ethics committee of the Landesärztekammer Hamburg (Medical Association of Hamburg, PV5131) approved the study protocol, and all participants provided informed consent. The study was registered at ClinicalTrial.gov (NCT03934957). All methods were performed in accordance with the relevant guidelines and regulations, including the Declaration of Helsinki and applicable national regulations.

Data collection and quality assurance of the HCHS are ongoing and we included data from the first 15,000 participants. Our analysis focused on employed individuals (full-time or part-time). Due to missing data, we conducted a complete-case analysis, resulting in a stepwise reduction of the available sample: 7,539 were employed, 5,822 had spirometry data; 5,263 had an occupational code; 5,124 had education levels recorded according to ISCED, 5,007 reported the number of years in their current occupation, 4,837 provided information on self-reported occupational exposure, and 4,811 had recorded smoking status This resulted in a final sample size of 4,811 for analyses using the GOLD-defined airway obstruction. For the GLI criteria, additional variables such as height and ethnicity were required, reducing the sample to 3,545 cases.

## Outcome variable: airway obstruction

In this study, we investigated the prevalence of airway obstruction at the population level. Spirometry was performed according to the criteria set by the European Respiratory Society (ERS) and the American Thoracic Society (ATS)^24^ as closely as possible. Typically, at least three spirometry attempts were performed for each participant to ensure accuracy. However, if a single high-quality attempt was obtained, as determined by trained study nurses, it was deemed sufficient. Participants were not administered any bronchodilators before undergoing the spirometry test but may have taken them independently prior.

Two sets of criteria were employed to diagnose an AO in this study:

1. GOLD Criteria: According to GOLD, airway obstruction is defined by a fixed ratio of the Forced Expiratory Volume in one second (FEV_1_) to the Forced Vital Capacity (FVC) below 0.7. FEV_1_is the volume of air a person can forcefully exhale in one second, while FVC is the total amount of air exhaled forcefully after taking a deep breath^21^.
2. GLI Criteria: GLI also assesses the FEV_1_/FVC ratio but use the ‘Lower Limit of Normal’ (LLN) as a reference. LLN is a statistical measure derived from healthy populations, tailored to specific age, gender, height, and ethnic groups, providing individualized benchmarks for normal lung function. Under the GLI criteria, an FEV_1_/FVC ratio falling below the individualized LLN indicates airway obstruction^22^.

We focused on airway obstruction rather than COPD to specifically identify the presence of spirometric obstruction, as a COPD diagnosis typically requires the presence of symptoms, exposure history, and post-bronchodilator obstruction, which was beyond the scope of our current analysis.

## Occupational exposure

We assessed exposure to VGDF in two distinct ways: through self-reporting and using a JEM. For self-reported exposure, we directly asked in the questionnaire: ‘Have you ever worked in a setting where you were exposed to vapors, gases, dusts, or fumes, thereby possibly inhaling hazardous substances?’ This question was designed to elicit a simple ‘yes’ or ‘no’ response, identifying participants who had potentially been exposed to VGDF in their workplace.

For the JEM-based assessment, we employed the Airborne Chemical Exposure Job-Exposure Matrix (ACE JEM). The ACE JEM was developed based on the UK SOC 2000 codes and categorizes exposures to VGDF across different occupations. To apply the ACE JEM to our German cohort, we mapped the German Classification of Occupations 2010 (KldB 2010) codes to the International Standard Classification of Occupations (ISCO-08), then to SOC 2010, and finally to SOC 2000 codes. This mapping process allowed us to utilize the ACE JEM for exposure assessment in our study. It is important to note that the mapping of occupational classifications inherently involves a degree of subjective judgement, which can introduce potential errors. To mitigate these, difficult cases were discussed to ensure a consensus was reached. Additionally, there was a final verification step to ensure that the occupations as defined by SOC 2000 remained consistent with those in KldB 2010.

The ACE JEM was selected due to its comprehensive coverage of airborne chemical exposures relevant to our research question and because there is no German equivalent. The ACE JEM assigns exposure levels to each of the 353 SOC codes based on data from various sources, including expert judgments and consensus discussions, considering typical work routines. Each occupation is categorized with a binary ‘yes’ or ‘no’ for occupational exposure to VGDF, based on the level of exposure for each noxious agent (low, medium, high), and the proportion of workers likely to be exposed to each agent (< 5%, 5–19%, 20–49%, ≥ 50%). The matrix does not account for accidental exposures or seasonal variations. A detailed description of the ACE JEM can be found in Sadhra et al.^25^.

## Covariates

Age, gender, and educational status (categorized as low, medium, and high according to the International Standard Classification of Education, ISCED) were analyzed^26^. Age was categorized into two groups: 45–54 years and 55 years and older. This categorization was chosen to facilitate data interpretation and to create two approximately equal-sized groups (46.1% and 53.9% of the sample). Smoking status was categorized as ‘never smoker’, ‘ex-smoker for at least six months’, and ‘currently smoker’. Participants reported their employment status (full-time or part-time) and years in their current occupation. Duration in the current occupation was categorized into three groups to represent different levels of exposure duration: short-term (≤ 3 years), medium-term (4–<10 years), and long-term (≥ 10 years). Occupational skill level, derived from KldB 2010 and categorized into Skill Level 1 (lowest complexity) to Skill Level 4 (highest complexity), was also included^27^.

## Statistical analyses

For ordinal variables, such as educational level and occupational status, descriptive statistics, including counts and percentages, were calculated. Similarly, for metric variables such as age and years in the current occupation, we computed means and standard deviations (SD). Cohen’s Kappa was calculated to assess the inter-rater reliability between self-reported and JEM-based occupational exposure assessments. Logistic regression models, both crude and adjusted, were used to examine the association between AO as dependent variable (according to GOLD and GLI criteria, with no AO as the reference) and occupational exposure (self-reported and JEM, with not exposed as the reference). Covariates included in the adjusted logistic regression models were selected based on their known associations with both occupational exposure and airway obstruction, as identified in the literature. Odds ratios (ORs) and 95% confidence intervals were calculated, with adjustments for age (≥ 55 vs. 45–54 as reference), gender (male vs. female as reference), education (ISCED levels, with the highest level as reference), occupational status (part-time vs. full-time as reference), years in current job (≤ 3 years as reference), and smoking status (never-smoker as reference). Although the GLI criteria adjust for age and sex in calculating the LLN, we included age and sex as covariates in our regression models to adjust for any residual confounding and to assess their independent associations with airway obstruction. This approach ensures consistency across analyses using both GOLD and GLI criteria and allows for direct comparison between models. We used standard thresholds to indicate statistical significance: *p* < 0.05 (*), *p* < 0.01 (**), and *p*< 0.001 (***) All analyses were conducted using R software, version 4.3.1^28^, with GLI LLN-reference values obtained from the ‘rspiro’ package^29^.

## Results

In our study of 4,811 participants, demographic and clinical characteristics showed notable gender differences (Table 1). The mean age was 55.9 years, with a higher representation of men (53.5%) than women (46.5%). Educational levels varied, with 60.8% of men achieving ‘High’ level versus 49.2% of women. Part-time employment was more common among women. Smoking status was similarly distributed between genders, with a slightly higher proportion of current smokers among men. Clinically, the prevalence of airway obstruction varied by diagnostic criteria and gender: AO prevalence was higher in men (15.5%) compared to women (13.4%) when assessed using the GOLD criteria, whereas the GLI criteria showed a higher prevalence in women (7.5%) compared to men (6.9%). For occupational exposure to noxious substances, men had higher prevalence. However, JEM assessments indicated an increase in the proportion of exposed individuals in both genders, unlike self-reported exposure.

**Table 1:** Characteristics of the study population, stratified by gender. (SD = standard deviation; ISCED = International Standard Classification of Education; AO = airway obstruction; GOLD = Global Initiative for Chronic Obstructive Lung Disease; GLI = Global Lung Function Initiative).

	Women (*n* = 2238)	Men (*n* = 2573)	Total (*n* = 4811)
Age			
-Mean (SD)	55.4 (5.6)	56.3 (6.3)	55.9 (6.0)
Age in Categories—*n* (%)			
−45–54	1078 (48.2)	1139 (44.3)	2217 (46.1)
-≥55	1160 (51.8)	1434 (55.7)	2594 (53.9)
ISCED-Education—*n* (%)			
-High	1101 (49.2)	1565 (60.8)	2666 (55.4)
-Medium	1086 (48.5)	940 (36.5)	2026 (42.1)
-Low	51 (2.3)	68 (2.6)	119 (2.5)
Skill Level (low to high)—*n* (%)			
-Category 1	73 (3.3)	34 (1.3)	107 (2.2)
-Category 2	993 (44.4)	829 (32.2)	1822 (37.9)
-Category 3	470 (21.0)	566 (22.0)	1036 (21.5)
-Category 4	702 (31.4)	1144 (44.5)	1846 (38.4)
Employment status—*n* (%)			
-Full-time	1234 (55.1)	2326 (90.4)	3560 (74.0)
-Part-time	1004 (44.9)	247 (9.6)	1251 (26.0)
Years in current occupation—*n* (%)			
-≤ 3	141 (6.3)	154 (6.0)	295 (6.1)
−4 - <10	383 (17.1)	376 (14.6)	759 (15.8)
-≥ 10	1714 (76.6)	2043 (79.4)	3757 (78.1)
Smoking status—*n* (%)			
-Never-smoker	884 (39.5)	911 (35.4)	1795 (37.3)
-Ex-Smoker	818 (36.6)	1026 (39.9)	1844 (38.3)
-Smoker	536 (23.9)	636 (24.7)	1172 (24.4)
GOLD-AO—*n* (%)			
-No	1939 (86.6)	2173 (84.5)	4112 (85.5)
-Yes	299 (13.4)	400 (15.5)	699 (14.5)
GLI-AO—*n* (%)			
-No	1544 (92.5)	1745 (93.1)	3289 (92.8)
-Yes	126 (7.5)	130 (6.9)	256 (7.2)
-*n* miss^1^	568	698	1266
Self-reported occupational exposure—*n* (%)			
-Not exposed	1937 (86.6)	1821 (70.8)	3758 (78.1)
-Exposed	301 (13.4)	752 (29.2)	1053 (21.9)
Job-Exposure Matrix exposure—*n* (%)			
-Not exposed	1716 (76.7)	1627 (63.2)	3343 (69.5)
-Exposed	522 (23.3)	946 (36.8)	1468 (30.5)

^1^missings due GLI-AO criteria.

Table 2a provides a comparative analysis of occupational exposure to inhalable noxious substances, contrasting self-reported data with findings from the ACE JEM. In our study population, 12.3% of the participants were classified as exposed by both self-reporting and JEM, while 59.9% were classified as not exposed by both methods. The inter-rater reliability between self-reported and JEM-based exposure, measured by Cohen’s kappa, was 0.29, indicating fair agreement. Table 2b further refines this analysis by evaluating the agreement between self-reported exposure and JEM across skill levels. It reveals a pattern: workers at the lowest and highest ends of the job qualification spectrum have comparably poor exposure awareness.

**Table 2:** (**a**) Cross-table between occupational exposure to inhalable noxious substances according to self-report in the questionnaire and the Job-Exposure matrix (JEM), *n* = 4811. (**b**) comparison of occupational exposure assessment: Self-Reported versus Job-Exposure matrix by occupational requirement level.

**(a)**			
	JEM exposure		
	Exposed	Not exposed	Total self-reported
Exposed	591 (12.3%)	462 (9.6%)	1053 (21.9%)
Not exposed	877 (18.2%)	2881 (59.9%)	3758 (78.1%)
Total JEM	1468 (30.5%)	3343 (69.5%)	4811 (100%)
Cohen‘s Kappa 0.29			

**(b)**					
Skill Level (low to high)	Self-Reported		JEM		Cohen’s Kappa
	*n* exposed	*n* not exposed	*n* exposed	*n* not exposed	
Category 1	30	77	77	30	0.17
Category 2	502	1320	719	1103	0.33
Category 3	242	794	300	736	0.33
Category 4	279	1567	372	1474	0.14

Our study’s findings, as detailed in Tables 3 and 4, delve into the relationship between occupational exposure to VGDF and AO, evaluated through two diagnostic criteria: GOLD and GLI. These analyses incorporate occupational exposures as assessed both by self-report and through the JEM.

In Table 3, focusing on GOLD-defined AO, a modest increase in AO risk associated with occupational exposure is observed. The odds ratios for the crude model were 1.14 (95% CI 0.94–1.38) for self-reported exposure and 1.11 (95% CI 0.94–1.32) for JEM-based exposure. However, these associations slightly diminish in the adjusted models, resulting in ORs of 1.08 (95% CI 0.88–1.30) for self-reported exposure and 1.06 (95% CI 0.89–1.27) for JEM-based exposure, after controlling for age, gender, education, employment status, years in current occupation, and smoking status.

**Table 3:** Logistic regression analyses of the association between occupational exposure to vapors, gases, dusts, and fumes (VGDF) and GOLD-defined airway obstruction (AO) (Ref. No AO), assessed via self-report and the Job-Exposure matrix (JEM). Odds ratios with 95% confidence intervals. (ISCED = International Standard Classification of Education; AO = airway obstruction; GOLD = Global Initiative for Chronic Obstructive Lung Disease).

	Self-reported		Job-Exposure Matrix	
	unadjusted (1)	adjusted (2)	unadjusted (3)	adjusted (4)
Occupational exposure (ref. not exposed)	1.14	1.08	1.11	1.06
	(0.94–1.38)	(0.88–1.30)	(0.94–1.32)	(0.89–1.27)
Age 55 and older (ref. 45–54)		1.43 ***		1.42 ***
		(1.21–1.70)		(1.20–1.68)
Gender male (ref. female)		1.20 *		1.21 *
		(1.00–1.44)		(1.01–1.45)
ISCED Medium (ref. high)		0.90		0.90
		(0.76–1.07)		(0.76–1.07)
ISCED Low		0.99		0.98
		(0.58–1.60)		(0.57–1.58)
Part-time occupation (ref. full-time)		1.18		1.18
		(0.97–1.44)		(0.97–1.44)
4–10 years in current occupation (ref. ≤ 3)		0.96		0.96
		(0.66–1.41)		(0.66–1.41)
> 10 years in current occupation		0.86		0.86
		(0.62–1.21)		(0.62–1.21)
Smoke status ex-smoker (ref. never-smoker)		1.59 ***		1.59 ***
		(1.30–1.95)		(1.30–1.95)
Smoke status smoker		2.26 ***		2.27 ***
		(1.83–2.80)		(1.83–2.81)
Number of Observations	4,811	4,811	4,811	4,811
Akaike Information Criterion	3990.0	3922.6	3990.4	3922.7

* *p* < 0.05, ** *p* < 0.01, *** *p* < 0.001.

Table 4, which assesses GLI-defined AO, indicates a stronger association with occupational exposure in the self-reported data. The unadjusted model for self-reported exposure revealed a significant increase in the likelihood of GLI-AO (OR 1.48; 95% CI 1.11–1.96). This significant association persists in the adjusted model (OR 1.42; 95% CI 1.05–1.90). In contrast, the JEM-based exposure models did not show statistically significant associations in both unadjusted (OR 1.06; 95% CI 0.80–1.38) and adjusted analyses (OR 1.02; 95% CI 0.77–1.36).

**Table 4:** Logistic regression analyses of the association between occupational exposure to vapors, gases, dusts, and fumes (VGDF) and GLI-defined airway obstruction (AO) (Ref. No AO), assessed via self-report and to Job-Exposure matrix (JEM). Odds ratios with 95% confidence intervals. (ISCED = International Standard Classification of Education; AO = airway obstruction; GLI = Global Lung Function Initiative).

	Self-reported		Job-Exposure Matrix	
	unadjusted (1)	adjusted (2)	unadjusted (3)	adjusted (4)
Occupational exposure (ref. not exposed)	1.48 **	1.42 *	1.06	1.02
	(1.11–1.96)	(1.05–1.90)	(0.80–1.38)	(0.77–1.36)
Age 55 and older (ref. 45–54)		0.84		0.82
		(0.65–1.09)		(0.63–1.07)
Gender male (ref. female)		0.90		0.96
		(0.68–1.21)		(0.72–1.28)
ISCED Medium (ref. high)		1.20		1.22
		(0.92–1.56)		(0.94–1.60)
ISCED Low		0.89		0.93
		(0.34–1.94)		(0.35–2.06)
Part-time occupation (ref. full-time)		1.09		1.08
		(0.79–1.48)		(0.79–1.47)
4–10 years in current occupation (ref. ≤ 3)		0.80		0.81
		(0.48–1.37)		(0.48–1.38)
> 10 years in current occupation		0.61 *		0.62 *
		(0.39–0.99)		(0.40–0.99)
Smoke status ex-smoker (ref. never-smoker)		1.42 *		1.44 *
		(1.02–2.00)		(1.03–2.03)
Smoke status smoker		2.78 ***		2.83 ***
		(2.00–3.91)		(2.03–3.98)
Number of Observations	3,545	3,545	3,545	3,545
Akaike Information Criterion	1835.6	1798.9	1842.5	1804.0

* *p* < 0.05, ** *p* < 0.01, *** *p* < 0.001.

**Fig. 1:**
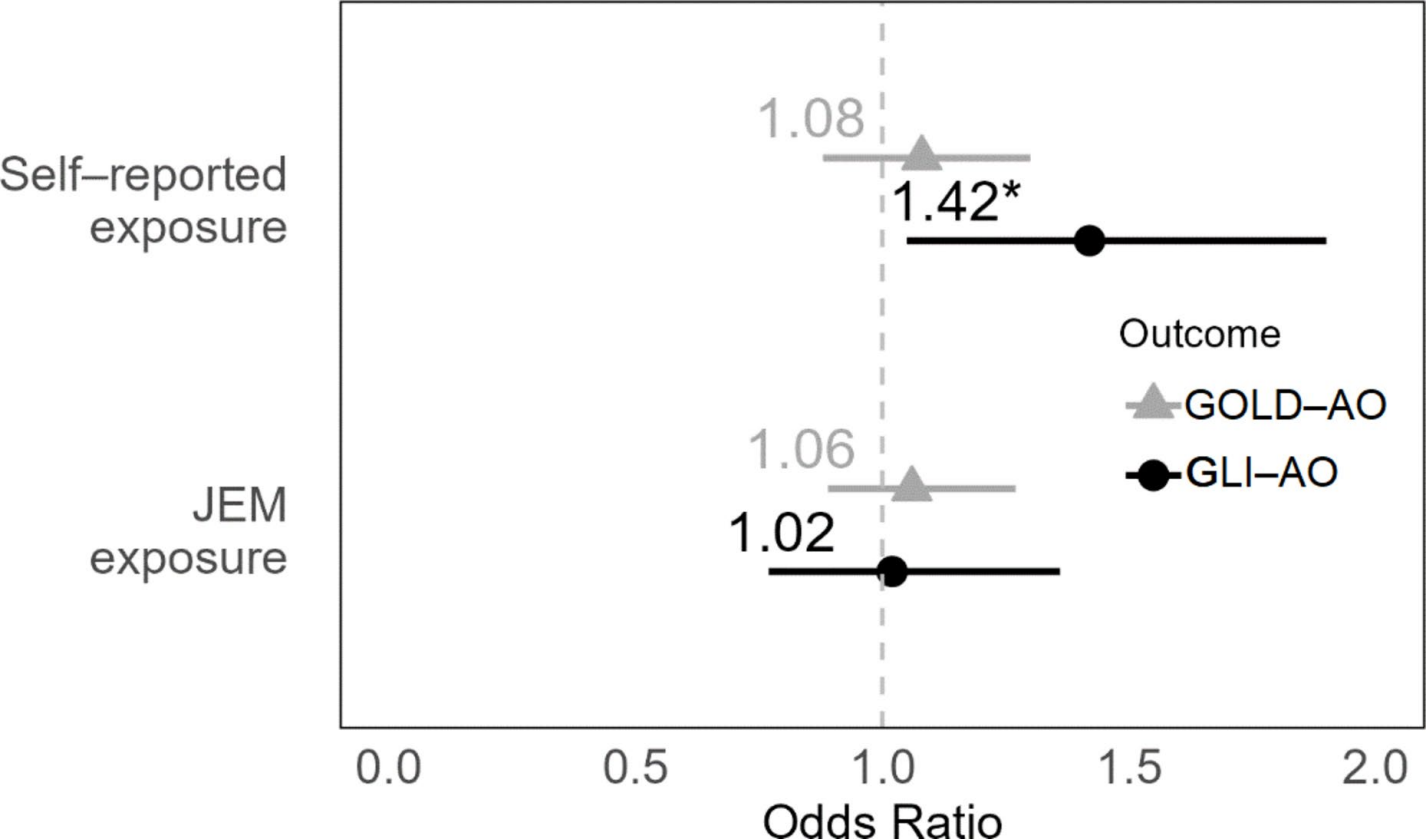
Visual representation of the main results of the four adjusted logistic regression models in Tables 3 and 4 in an interval plot. Adjusted for age, gender, education, occupational status, years in occupation and smoking status. (JEM = Job-Exposure Matrix; GOLD = Global Initiative for Chronic Obstructive Lung Disease; GLI = The Global Lung Function Initiative; AO = airway obstruction). * p < 0.05, ** p < 0.01, *** p < 0.001.

Figure 1, an interval plot, illustrates the logistic regression models’ results, showing the relationship between occupational exposure and AO using both GOLD and GLI criteria. The plot highlights different outcomes for self-reported exposure: the OR for GOLD-AO is 1.14 (non-significant), while the OR for GLI-AO is 1.48 (95% CI 1.11–1.96), indicating a significant association. In contrast, JEM-based exposure shows an OR of 1.11 for GOLD-AO and 1.06 for GLI-AO, with neither reaching statistical significance.

## Discussion

The findings of this study shed new light on the complex relationship between occupational exposure to VGDF and the development of an airway obstruction, as delineated by the GOLD and GLI criteria. Our study reveals a particular low occupational exposure recognition for workers with the highest and lowest skill levels, suggesting potential underreporting in these groups. Furthermore, the associations observed were modest in the context of GOLD-defined airway obstruction, self-reported exposure demonstrated a more pronounced link with GLI-defined AO. This discrepancy underscores the importance of considering different diagnostic criteria and methods of exposure assessment in understanding the occupational risks associated with AO. The distinction between self-reported and JEM-based exposure assessments further emphasizes the need for a multifaceted approach in evaluating occupational health risks.

To accomplish this in clinical or research settings, combining multiple assessment methods can enhance the accuracy and reliability of exposure evaluations. For instance, integrating self-reported exposure data with JEM assessments allows for cross-verification and helps to identify discrepancies or underreporting. Additionally, incorporating direct exposure measurements, such as environmental monitoring or biomarker analysis, can provide objective data to supplement subjective reports. In clinical practice, detailed occupational histories taken by healthcare professionals can be augmented with expert assessments from occupational hygienists. An interdisciplinary approach that includes epidemiologists, industrial hygienists, and clinicians can facilitate the use of comprehensive exposure assessment tools. By leveraging the strengths of various methods, a multifaceted approach can provide a more accurate representation of occupational exposures, ultimately leading to better risk assessment and more effective prevention strategies.

The concordance between self-reported occupational exposure and JEM-derived exposure was modest. These findings align with similar inter-rater reliability reported in the literature. For instance, Blanc et al. observed a Kappa of 0.37 when comparing a single VGDF item with the Asthma JEM. Quinlan reported a Kappa of 0.35 comparing a single VGDF item with a JEM^30^. Koch et al. reported a Kappa of only 0.17 when using a smaller sample of the HCHS and a subset of health care and welfare workers^31^. The concept of risk homeostasis in occupational health psychology might offer insights into why workers may underreport their exposure prevalence compared with a JEM^32^. This theory argues that individuals tend to accept a certain level of risk in their environment, adjusting their behaviour based on the perceived level of risk. In occupational settings, workers often accept the risks associated with VGDF exposure, leading to underreporting in self-assessments. This acceptance could be a result of long-term adaptation to workplace conditions, a lack of adequate training about hazards, or a workplace culture that downplays risks.

The potential underreporting of exposures at both the lowest and highest skill levels may be influenced by a range of factors. At lower skill levels, workers may underreport exposures due to a lack of hazard awareness, limited safety training, or an acceptance of risk as an inherent part of their job. This normalization of risk could lead them to perceive hazardous conditions as routine and not worth reporting. In contrast, workers at higher skill levels might underreport exposures because they overestimate their ability to control or mitigate risks. This overconfidence could result in a perception of being less vulnerable to occupational hazards, leading to an underestimation of actual exposure levels. Additionally, workers in higher-skilled roles might operate in environments perceived as safer or engage in tasks that obscure their actual levels of exposure.

Our findings align with previous research on the associations between self-reported and JEM assessed VGDF exposure with COPD risk. In a meta-analysis, for instance, Sadhra et al.^33^demonstrated in a meta-analysis that both, self-reported and JEM-based exposures to VGDF are associated with doctor-diagnosed COPD risk. When considering spirometry assessed COPD Sadhra et al. found however, that self-reported, but not JEM assessed, exposure to VGDF was associated with an elevated risk of COPD. More recent analyses from the UK Biobank, which also applied the ACE JEM, reported a comparable prevalence of VGDF exposure (33.5–35.5%) to that observed in our cohort (36.8%). In contrast to our findings, these analyses identified a slight but significant association with airflow obstruction (adjusted prevalence ratio: 1.04; 95% CI: 1.01–1.07)^34^. Notably, when the UK Biobank applied the ALOHA + JEM and incorporated lifetime occupational histories, no association between VGDF exposure and COPD was detected^35^.

The variance in findings, particularly the lower risk estimators for JEM-assessed COPD risk exposures compared to self-reported exposures, invites a multifaceted exploration. One possible explanation for this pattern could be the intrinsic nature of JEMs to generalize exposure across job categories, potentially diluting individual variations in exposure levels that might be more accurately captured in self-reported data. This generalization can lead to an underestimation of risk in diverse populations where exposure intensity varies significantly within the similar jobs. Furthermore, discrepancies in study designs, such as differences in exposure duration assessment, latency since cessation of an exposure, outcome assessment, variability in the specific questions asked in self-report questionnaires, and the criteria for categorizing exposure in JEMs, could contribute to these inconsistencies. Additionally, self-reported data can be subject to recall bias and reporting inaccuracies, as individuals may not accurately remember or may misreport their exposure levels. Such methodological diversity highlights the complexity of occupational exposure assessment and the need for more standardized and sensitive approaches to assess and interpret the relationship between occupational exposure and COPD risk.

It is anticipated that the risk of COPD increases with higher age, which may reflect the cumulative effect of various risk factors over time^22^. This expectation aligns with the conventional understanding that prolonged exposure to respiratory irritants, combined with age-related physiological changes, increases COPD risk. In our study, this age-related risk escalation was evident for GOLD-defined airway obstruction, with older participants showing a significantly higher association (adjusted OR 1,24; 95% CI 1.20–1.68). However, under the GLI criteria, a divergent pattern emerged, where older participants had a lower, though not statistically significant, association of airway obstruction compared to younger participants (adjusted OR 0.82; 95% CI 0.63–1.07). This unexpected finding mirrored a similar age trend observed in only one other study^36^. The GLI reference values incorporate age as a variable, which adjusts predicted lung function and reduces the likelihood of older adults falling below the LLN. Despite this adjustment, COPD prevalence and risk typically increase with age, and the absence of this trend in our findings under GLI criteria is unusual and merits further investigation.

Different patterns of occupational exposure of varying latency periods after exposure cessation may also contribute to these age-related differences. However, the lack of consistent patterns between AO defined by GOLD and GLI criteria suggests that these findings are not solely due to selection bias. Instead, the results highlight critical questions about the universality and applicability of these reference standards, particularly for specific population with diverse health profiles and environmental exposures.

Another interesting aspect of our findings is the identification of tenure exceeding 10 years in the current occupation as a statistically significant protective factor against AO as defined by GLI. This result was unobserved under the GOLD criteria as the prevalence of AO generally increases with age using this criterion. This distinction in the GLI-based evaluation suggests potential biases in occupational health studies, specifically the Healthy Worker Bias and survivorship bias. The Healthy Worker Bias posits that actively employed individuals tend to have better health profiles than the general population from the start, primarily because those with significant health issues are less likely to enter or remain employed^37^. Similarly, survivorship bias may play a role, where individuals who have managed to stay employed for over a decade are likely those who have not developed severe health conditions such as COPD, possibly due to less exposure to high-risk occupational factors or inherently better health. Conversely, those who develop health complications might be compelled to leave their jobs earlier, thus skewing the perceived risk factors in long-term employees^38^. This results in potential underestimation of the true long-term risks and prevalence of conditions like COPD among workers.

This study is the first examination of occupational exposure to VGDF in a German population cohort using both self-reported assessment and a JEM while distinctively evaluating the association of these exposures with airway obstruction by applying two separate diagnostic criteria: GOLD and GLI parameters. This dual-method approach for both exposure assessment and AO outcome measurement marks a novel contribution to the field. Incorporated within the framework of the HCHS cohort, this investigation leverages a substantial sample size and rigorous methodology. Trained professionals conduct spirometry, mostly adhering to the ATS/ERS criteria, and ensuring the reliability and accuracy of respiratory functional measurements.

However, several limitations are noteworthy. First, although participants were randomly selected from the residents’ registration office, the individuals who chose to participate may not be fully representative of the general population. Specifically, there was a higher proportion of participants with higher educational levels, particularly among men. This overrepresentation of individuals with an advanced socioeconomic position may affect the prevalence estimates and the observed associations, potentially limiting the generalizability of our findings. Second, people with functional limitations might have faced difficulties in accessing our study centre.

Third, another limitation of our study is the presence of missing data, which could potentially lead to bias if the missingness is not at random. While some missing data likely resulted from random factors such as equipment malfunctions (e.g., spirometry device issues) or oversight during data collection (e.g., unmeasured body height), it is possible that other missing data may be related to participant characteristics. For instance, participants in lower-skilled occupations might have been less inclined to report their job titles or occupational histories, possibly due to concerns about confidentiality or perceived stigma. If the likelihood of missing data is associated with both exposure and outcome, this could introduce non-random missingness and bias our results.

Fourth, occupational classifications were derived from questionnaire data, with an inherent risk of misclassification. We have applied the ACE-JEM which was developed for categorising jobs in the UK and which demonstrated a notable degree of concordance in categorizing jobs with ‚high‘ and ‚no‘ exposure in an evaluation study^39^. It may, however, be less effective in categorizing jobs with ‘low’ and ‘medium’ exposure. Fifth, we acknowledge that our self-reported exposure assessment is limited to ‘ever’ exposure, which does not account for the intensity, frequency, or duration of exposure to VGDF. Sixth, although we collected questionnaire data on medically diagnosed asthma and chronic bronchitis/COPD, we did not adjust for these pre-existing respiratory conditions in our analyses. Our focus was on spirometry-defined airway obstruction to provide an objective assessment of lung function. Seventh, an important limitation is our inability to adjust for smoking intensity and duration (e.g., pack-years) due to a high proportion of missing data. We adjusted for smoking status (never, former, current smoker), but this may not fully account for the effects of smoking. Eighth, the urban sample limits the generalization of our findings to more rural populations. Therefore, future research should consider regional differences in occupational exposures to assess the external validity of these findings in different contexts.

## Conclusion

The findings of this study illuminate the complex relationship between occupational exposure to VGDF and development of airway obstruction, as per GOLD and GLI criteria. Our study identifies a discrepancy in occupational COPD risk recognition between the highest and lowest skill levels, suggesting potential underreporting. While the associations were modest for GOLD-defined AO, self-reported exposure showed a stronger link with GLI-defined AO. This discrepancy highlights the importance of considering different diagnostic criteria and exposure assessment methods in understanding risks for occupational diseases with airway obstruction like COPD. The differences based on GOLD and GLI criteria emphasize the need for further research to understand these discrepancies and to develop better occupational health policies. Our findings underscore the importance of tailoring public health interventions and occupational safety regulations to workers’ specific needs and occupational risks. Targeted approaches are essential for effectively mitigating the risk of occupational diseases such as COPD and for ensuring the health and safety at work.

## Data Availability

Pseudonymized data used in this study can be accessed by interested researchers upon reasonable request to the HCHS Steering Committee, subject to approval and the completion of a material transfer agreement.
